# Physical Fitness after Anterior Cruciate Ligament Reconstruction: Influence of Graft, Age, and Sex

**DOI:** 10.3390/sports8030030

**Published:** 2020-03-06

**Authors:** Robert Csapo, Helmut Pointner, Christian Hoser, Peter Gföller, Christian Raschner, Christian Fink

**Affiliations:** 1 Research Unit for Orthopaedic Sports Medicine and Injury Prevention, Institute for Sports Medicine, Alpine Medicine & Health Tourism (ISAG), UMIT-Private University for Health Sciences, Medical Informatics and Technology, Eduard-Wallnöfer-Zentrum 1, 6060 Hall, Austria; c.hoser@gelenkpunkt.com (C.H.); p.gfoeller@gelenkpunkt.com (P.G.); c.fink@gelenkpunkt.com (C.F.); 2 Sporttherapie Huber & Mair–Private Practice, Steinbockallee 31, 6063 Rum, Austria; spowi@sporttherapie-hm.at; 3 Gelenkpunkt–Sports and Joint Surgery, Olympiastraße 39, 6020 Innsbruck, Austria; 4 Department of Sport Science, University of Innsbruck, Fürstenweg 185, 6020 Innsbruck, Austria; christian.raschner@uibk.ac.at

**Keywords:** anterior cruciate ligament (ACL) reconstruction, physical fitness, strength, agility, jump performance, return-to-sports, back in action

## Abstract

Functional tests are used to facilitate return-to-sports decisions after anterior cruciate ligament reconstruction (ACLR). This study presents comprehensive physical fitness test data acquired in highly active patients within the first year after ACLR, for comparison between different grafts, age groups, and sexes. The outcomes from a specific seven-item test battery and isokinetic strength test data were extracted from a patient database. Results were compared to normative data from age- and sex-matched controls and between subgroups of patients. A total of 245 patients (94 women, 23.8 ± 8.4 years, pre-injury Tegner 7.4 ± 1.6) were tested 185 ± 44 days after surgery. In 116 patients (47.3%), one or more test results were classified as “poor” or “very poor” after comparison with normative data, with failures being most frequent during single-leg squat jump and plyometric strength tests. Test failures were more prevalent in adults than in adolescents <19 years (61.4%–62.2% vs. 24.5%, *p* < 0.001) and in men (61.6% vs. 24.5%, *p* < 0.001), but no differences were found between grafts. Isokinetic knee extensor strength was lower by 24.1% on the injured side. Six months after ACLR, nearly 50% of highly active patients presented with strength and functional fitness deficits. These deficits are particularly prevalent in older patients and men.

## 1. Introduction

Ruptures of the anterior cruciate ligament (ACL) represent one of the most common musculoskeletal injuries and typically affect young and athletic populations [1,2]. In patients desiring to return to sports—particularly level I sports involving jumping, pivoting, and cutting maneuvers—reconstruction of the torn ligament (ACLR) is usually the only viable treatment option to restore adequate joint stability. Such interventions notwithstanding, only 19%–48% of athletes succeed in returning to sports within 12 months [3] and the risk of graft failures or secondary ACL injuries on the contralateral limb is high [4], especially in pediatric cohorts [5].

In the light of the high reinjury rates in athletes, the timing of the return-to-sports (RTS) is of crucial importance. While often solely based on the time after surgery [6], recent years have seen significant efforts in the development of objective criteria to facilitate RTS decisions [7,8,9,10]. One core component of such criteria is the physical performance in different functional tests. A current meta-analysis including a total of five studies concluded that passing RTS criteria reduced the risk of subsequent graft rupture by 60%, while increasing that of contralateral ACL rupture by 235% [11]. However, large-scale randomized prospective studies comparing cohorts matched for potentially influencing factors, such as age, sex, athletic discipline, or performance level are missing, therefore care must be taken when interpreting these results.

The wide variety of tests proposed indicates that no consensus exists regarding the specific measures to be included into RTS test batteries. The most commonly reported characteristic is hop test performance [7], but test series may also include strength or agility tests as well as measures of movement quality. Comprehensive test batteries are not without controversy, as factor analyses suggest that the results of several frequently performed tests may share a large amount of variance [12], rendering them partly redundant. Moreover, the interpretation of test results is complicated, as the rehabilitation progress may be influenced by graft selection as well as the patients’ age, sex, and physical activity level. Although several cross-sectional studies have analyzed thigh muscle strength deficits in patients treated with different grafts [13,14,15], detailed comparisons of physical performance profiles of different populations of patients recovering from ACLR are missing.

Several years ago, we developed a standardized series of functional tests that became known as the *Back in Action* (BIA; CoRehab srl, Trento, Italy) test battery [16]. The battery consists of a total of seven tests, including uni- and bilateral balance tests, countermovement and plyometric jumps, agility tests, and measurements of cyclic movement velocity. Normative data acquired in over 400 participants allow for the comparison of results with age- and sex-matched healthy control subjects. Since its introduction in 2014, the BIA test battery has been administered to approximately 250 physically active subjects on at least one occasion after ACLR. The main aims of this report are to: (i) present the BIA and additional strength test data acquired in these patients; (ii) compare test results between patients treated with different grafts, age groups, and sexes; and (iii) identify key functional deficits.

## 2. Materials and Methods

### 2.1. Design

The database of a specialized sports clinic was retrospectively screened for patients who had undergone the BIA test series within the first 12 months after ACLR. In addition to BIA and strength test results, extracted data included the patients’ sex and age, the time with respect to the date of surgery at which tests were performed, and the kind of graft used. Considering their small number, patients receiving patellar tendon grafts (*n* = 5) were excluded. Patient-reported outcome measures including subjective ratings of pain (measured on a visual analog scale), as well as Lysholm [17] and Tegner activity scores [18]—reflecting the state before injury and at 6-months follow-up—as well as the main sports disciplines in which subjects indicated to be mostly active were also extracted for a more comprehensive presentation of the study sample.

### 2.2. Patients

A total of 245 patients (151 males, 61.6%; 94 females, 38.4%) met our criteria for inclusion. On average, patients were 23.8 ± 8.4 years old, with 14 (5.7%), 80 (32.7%), 114 (46.5%), and 37 (15.1%) aged between 10–14, 15–19, 20–29, and 30–50 years, respectively. Subjects indicated to be predominantly active in one of 32 different sports disciplines, with alpine skiing (*n* = 97, 39.6%), soccer (*n* = 64, 26.1%), and recreational fitness training (*n* = 24, 9.8%) being most frequently mentioned. Detailed information on the injuries suffered and the graft used were available in 227 cases. In the majority of these (139, 61.2%), quadriceps (QT) tendon autografts were implanted. In 88 (38.8%) cases, hamstring (gracilis or semitendinosus, SGT) tendons were used. The surgeries consisted of 183 primary ACLR (80.6%), 24 revisions (10.6%), and 20 ACLR after previous ACL rupture on the contralateral limb (8.8%). Injuries were classified as simple and complex (involvement of medial or lateral meniscal tears or chondral lesions) in 110 (48.5%) and 117 (51.5%) cases, respectively. The analysis of data was approved by the Medical University of Innsbruck ethics committee (AN2016-0067). The sample’s main characteristics are summarized in Table 1.

### 2.3. Procedures

The BIA data comprise the results of the following seven tests: two-leg stability and single-leg stability on an unstable platform; two-leg and single-leg countermovement jump; plyometric reactive strength index test; “Speedy” jump coordination test; and the “Quick feet” agility test. All single-leg tests were performed with both the injured and non-injured leg and for jumps both maximum jump height and power were calculated, yielding a total of 13 outcome measures. A detailed description of the BIA test series and all included tests is available elsewhere [16]. The test outcomes were compared to age- (groups: 10–14, 15–19, 20–29, and 30–50 years) and sex-specific normative data, which were assessed in recreationally active subjects (2–3 days/week) engaging in a wide range of sports disciplines. For single-leg tests, normative data acquired in the dominant and non-dominant legs were compared to the non-injured and injured legs, respectively. Highly trained and professional athletes were deliberately excluded from the control group. Patients’ data were then classified as “very good” (1 SD above mean of normative data), “good” (0.5 SD above mean), “norm” (mean), “poor” (0.5 SD below mean) or “very poor” (1 SD below mean), respectively. “Poor” and “very poor” results were considered as test failures, which were summed for statistical comparison between groups. In addition, a cumulative BIA score was calculated, by assigning “very good” results the value of 1 and “very poor results” the value of 5. Hence, the cumulative BIA score may adopt values between 13–65, with lower values reflecting better overall performance.

In addition to BIA tests, patients were tested for isokinetic strength of the knee extensor and flexor muscles. Isokinetic strength measurements were performed in the same week as BIA tests and included four consecutive maximal knee extension/flexion cycles performed in a seated position from 10°–90° of knee flexion (0° representing the fully extended joint) at a velocity of 60 deg·s^−1^. The peak torques were extracted, averaged over the four consecutive trials and normalized to body mass, to obtain relative maximum voluntary contraction (MVC) knee extension and flexion torques, respectively (Nm·kg^−1^). Between-leg differences were calculated to quantify potential knee extensor or flexor strength deficits on the injured leg. All BIA and isokinetic tests were performed by a single, experienced examiner.

### 2.4. Statistical Analyses

For comparisons between age groups, children aged 10–14 years (*n* = 14) were pooled with adolescents aged 15–19 years (*n* = 80) to increase statistical power. Since Kolmogorov–Smirnov tests indicated a violation of the assumption of normality, Kruskal–Wallis (age groups) or Mann–Whitney U tests (grafts, sexes, post hoc pairwise tests) were used for between-group comparisons of patient-reported outcomes measures as well as the number of failed tests and the cumulative scores of BIA tests. The ratios of subjects failing one or more BIA tests were compared by Χ^2^ tests, and adjusted standardized residuals were calculated to follow-up significant results where appropriate. The results of isokinetic strength tests were normally distributed and, therefore, compared by one-way ANOVAs or independent samples *t*-tests, respectively. In case of significant results (*p* < 0.05), test statistics were converted into Pearson’s coefficients [19], which were reported as a measure of effect size. All statistical tests were performed using commercially available software (SPSS Statistics 25.0, IBM, Armonk, NY, USA).

## 3. Results

### 3.1. Patient-Reported Outcome Measures

Pre-injury levels of visual analog scale pain (VAS) pain were generally low and not statistically different between subgroups of patients receiving different grafts, age groups, or sexes (all *p* > 0.05). The slight increases observed from baseline to 6-months follow-up were not statistically different between subgroups of patients (all *p* > 0.05).

Pre-injury Lysholm scores did not differ between subgroups created by graft, age group, or sex. Changes in Lysholm scores from baseline to 6-months follow-up, however, differed significantly between age groups (H(2) = 9.963, *p* = 0.007), with reductions being smaller in 10–19 year old subjects as compared to older patients. Changes in Lysholm scores over time did not differ between grafts or sexes (both *p* > 0.05).

The pre-injury activity level, as reflected by Tegner scores, was higher in 10–19 and 20–29 year old subjects compared to 30–50 year old patients (H(2) = 33.416, *p* < 0.001; total *n* = 243). Changes in Tegner scores from baseline to 6-months follow-up did not differ between age groups (*p* > 0.05). Neither baseline values nor changes to 6-months follow-up differed between subgroups created by graft or sex (all *p* > 0.05). All pre-injury patient-reported outcome measures and the respective changes from baseline until the 6-months follow-up are summarized in Table 2

### 3.2. Back in Action Tests

On average, patients underwent the BIA test battery 185 ± 44 days after ACLR. A total of 129 patients (52.7%) passed all tests included in the series; whereas in 116 subjects (47.3%), the results achieved in one or more tests were classified as “poor” or “very poor” and, consequently, considered as test failures. On average, each subject failed 1.4 ± 2.2 tests and the cumulative BIA score, reflecting the performance in all tests, was 29.1 ± 9.9. The number of test failures was unevenly distributed across the BIA test items, with the measurements of single-leg jump height in the injured leg (24.5% failures) and of the plyometric reactive strength index (22.0%) being most frequently classified as “poor” or “very poor”. When only subjects passing these two tests were selected (*n* = 159), the percentage of test failures was reduced to 5% or less in all other tests. Table 3 provides an overview over the classifications of BIA test results.

Comparing the BIA performance across grafts, 51.1% and 45.5% of patients receiving QT and SGT autografts, respectively, failed one or more BIA tests. Neither the test clearance ratios, average number of test failures, nor the cumulative BIA score were significantly different between grafts (all *p* > 0.05).

Comparisons of age groups showed that 24.5%, 61.4%, and 62.2% of subjects aged 10–19, 20–29, and 30–50 years, respectively, failed one or more BIA tests. These differences in failure rates were statistically significant (Χ^2^(2) = 32.030, *p* < 0.001). Analyses of adjusted standardized residuals showed that the test failure rate was significantly lower than expected in those aged 10–19 years (z = −5.7, *p* < 0.001), but significantly higher in the 20–29-year-old patients (z = 4.1, *p* < 0.001). Among subjects aged 20 years or older (*n* = 151), 33.1% of test failures were recorded in both the single-leg jump height test in the injured leg and the plyometric reactive strength index test. In contrast, only 10.6% and 4.3% of those aged 19 years or younger failed in these two most challenging tests. Statistically significant differences between age groups were also found in the average number of failed tests (H(2) = 43.698, *p* < 0.001). Post hoc pairwise comparisons showed that the 10–19-year-old subjects (0.33 ± 0.65 failed tests) failed significantly less tests than those aged 20–29 years (2.2 ± 2.8 failed tests; *p* < 0.001, r = −0.43), and those aged 30–50 years (1.7 ± 1.7 failed tests; *p* < 0.001, r = −0.44). The cumulative BIA scores were 22.9 ± 5.6, 33.2 ± 10.6, and 32.6 ± 7.7 in the age groups of the 10–19, 20–29, and 30–50-year-olds, respectively. These differences were statistically significant (H(2) = 63.572, *p* < 0.001). Post hoc tests revealed significant differences between subjects aged 10–19 and 20–29 years (*p* < 0.001, r = −0.50), as well as between 10–19 and 30–50 years (*p* < 0.001, r = −0.53).

Test failure rates were also significantly different between sexes, with 61.6% of men but only 24.5% of women failing one or more BIA tests (Χ^2^(1) = 32.024, *p* < 0.001). On average men failed 1.95 ± 2.53 tests, in comparison to 0.54 ± 1.17 failures in women (U = 4265.5, *p* < 0.001, r = −0.36), and cumulative BIA scores were 31.9 ± 10.4 and 24.7 ± 6.9 in men and women, respectively (U = 4162, *p* < 0.001, r = −0.35).

### 3.3. Isokinetic Strength Tests

Isokinetic strength measurements were obtained in 242 subjects 182 ± 41 days after surgery. In the entire sample, relative knee extension MVC torques (Nm·kg^−1^) achieved with the injured leg were approximately 24.1% lower compared to the healthy leg. In comparison, relative knee flexion MVC torques were only 6.6% lower on the injured side. Both the relative strength deficits of the knee extensor (t(222) = 3.708, *p* < 0.001, r = 0.25) and the knee flexor muscles (t(222) = 4.357, *p* < 0.001, r = 0.29) were statistically different between grafts. Compared to QT, usage of SGT autografts was associated with significantly lower strength deficits of the knee extensors (−0.42 ± 0.35 Nm·kg^−1^ vs. −0.61 ± 0.39 Nm·kg^−1^; *p* = 0.001, r = 0.24) but greater relative weakness of the knee flexor muscles (−0.17 ± 0.21 Nm·kg^−1^ vs. -0.05 ± 0.18 Nm·kg^−1^).

**Figure 1:**
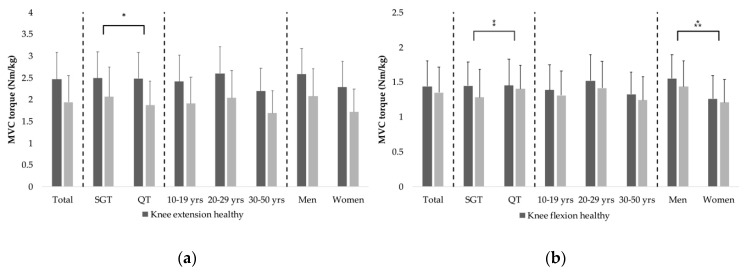
Isokinetic strength test data. Isokinetic strength tests of the knee extensor (**a**) and flexor (**b**) muscles. Bars and error bars show means and standard deviations of maximum torques normalized to body mass, respectively. * indicates the significantly greater knee extensor strength deficit of the injured leg in patients with a quadriceps tendon graft. ⁑ shows the significantly greater knee flexor strength deficit in patients with a hamstring tendon autograft. ⁂ denotes the significantly greater knee flexor strength deficit in men (all *p* < 0.05).

No significant differences in relative strength deficits on the injured leg were found between age groups (both *p* > 0.05). Between sexes, relative strength deficits of the knee extensors were not significantly different (*p* > 0.05). Relative strength deficits of the knee flexors, in contrast, were significantly greater in men (−0.12 ± 0.21 Nm·kg^−1^ vs. −0.05 ± 0.17 Nm·kg^−1^; t(221.6) = 2.503, *p* = 0.013, r = 0.16). The results of the isokinetic strength tests of the knee extensor and knee flexor muscles obtained in the entire sample as well as all subsets of patients are summarized in Figure 1.

## 4. Discussion

The present study aimed to investigate the performance level of patients recovering from ACLR surgery across a wide range of physical function tests, evaluate the influence of graft selection as well as the subjects’ sex and age, and identify key weaknesses of functional performance. Our data show that nearly 50% of patients failed in one or more BIA tests approximately 6 months after surgery. However, the tests included into this battery were found to be unequally challenging, with failure rates being highest in single-leg squat jumps performed with the injured leg and plyometric reactive strength tests. Between-group comparisons demonstrated that older patients and men struggled more to recover the average performance level of healthy control subjects matched for age and sex. Isokinetic tests further evidenced knee extensors strength deficits of nearly 25% in the injured leg, which were significantly greater in patients receiving QT compared to SGT autografts.

The BIA test battery consists of a series of seven tests developed with the intention to determine the physical readiness to return to sports after ACLR [16]. Including, power, speed, agility, and coordination tests, the BIA series was specifically designed to provide a comprehensive picture of different components of fitness that may be relevant for an individual’s capacity to return to sports. The first report about its clinical application presented data from 69 patients tested on average 170.7 and 239.1 days post-operatively [20]. The current study expands this earlier report by including data from a substantially larger sample and by evaluating the influence of the choice of ACL graft, sex, and age on test results.

Considering the high physical activity level of our sample (pre-injury Tegner 7.4 ± 1.6) and the fact that nearly 50% of patients failed in one or more tests 6 months after surgery, BIA must be considered a very challenging return-to-sports test. Thus, our results confirm an earlier report to demonstrate that BIA is more conservative compared to commonly performed strength and hop tests [21]. The test associated with the greatest failure rate was the single-leg countermovement jump performed with the injured leg, at which 60 out of 245 subjects (24.5%) achieved “poor” or even “very poor” results. Another test posing a great challenge was the plyometric reactive strength test, requiring patients to perform three consecutive jumps with both legs, aiming to maximize jump height while minimizing ground contact time. The finding that subjects passing both the single-leg countermovement jump and the plyometric reactive strength test also passed all other tests in 95% of cases clearly identifies these two tests as the most critical examinations. Isokinetic tests further revealed strength deficits of nearly 25% in the knee extensor muscles of the injured leg, confirming previous findings that show the recovery of knee extensor strength after ACLR occurs very slowly [22]. Considering that jump performance is known to be correlated to knee extensor strength [23], our results suggest that insufficiently recovered knee extensor strength is the key weakness of patients recovering from ACLR. Our findings therefore lend support to the recommendation by Barfod et al. [24] to include jump and knee extensor strength tests into RTS test series.

One important observation of our study was that both the number of failed tests and cumulative BIA scores reflecting overall test performance were affected by the patients’ age and sex, with functional deficits being significantly greater in older subjects and men. To date, the inter-individual differences in the recovery of physical fitness after ACLR have not been thoroughly studied. There is some evidence to suggest that knee extensor strength recovers more slowly in elderly women [25,26], but our isokinetic strength data do not support this hypothesis. Another study by Villa et al. [27] investigated factors influencing subjective International Knee Documentation Committee (IKDC) scores assessed during recovery from ACLR and found them to be significantly higher in younger patients, which lends partial support to our findings. Considering that not only pre-injury but also 6-months Tegner scores were greater in 10–19 year-old subjects compared to older subjects, it is plausible to assume that higher post-operative physical activity levels would benefit physical rehabilitation. In contrast, the reasons why men apparently struggle more than women to regain the average fitness level of age-matched controls cannot be explained by differences in physical activity levels, as neither pre-injury nor post-operative Tegner scores were statistically different between sexes. It could be that, irrespective of comparable activity levels, pre-injury physical fitness is higher in men, which would make it harder to regain that level. Also, it is possible that lower baseline fitness would predispose women to react more strongly to early-phase rehabilitation training. However, these explanations are highly speculative and require further investigation.

Further results that warrant discussion are the differences in the performance level of patients treated with different grafts. In agreement with previous studies reporting higher hamstrings-to-quadriceps strength ratios, when reconstruction was performed with either QT or patellar tendon as compared to SGT autografts [13,14,15], we found larger knee extensor but lower knee flexor strength deficits in patients with QT autografts. In contrast, performance in the more complex BIA tests was not significantly different between grafts. While the BIA tests involving jumps, which we found to be associated with the highest failure rates, do require sufficiently developed knee extensor strength and its recovery was found to proceed more slowly in patients with QT autografts, post hoc power analyses suggest that our study lacked statistical power to establish this relationship.

It is important to note some limitations of our study. First and foremost, the retrospective study design allowed us to gather data from a relatively large sample but complicated the tight control of the study population. While patient-reported outcome measures indicated comparable pre-injury levels of pain, knee, and age-associated differences in physical activity levels were documented, comparisons between groups (e.g., patients receiving different grafts) may still have been affected by a selection bias related to unknown factors (e.g., unequal severity of secondary injuries, such as meniscal tears). In addition, the evaluation of BIA test results needs to be mentioned. While the evaluation was based on the comparison with normative data acquired in healthy, physically active subjects (*n* > 400) of the same age and sex, our set of normative data does not necessarily reflect the average fitness levels of specific athletic cohorts. Hence, a test result classified as “Norm” may actually not suffice to warrant a safe RTS. Finally, all outcome measures reported were obtained approximately six months after ACLR. In the absence of follow-up measurements, these data do not allow for conclusions about the further time course of rehabilitation to be drawn.

## 5. Conclusions

Our data demonstrate that BIA testing identifies significant deficits in motor performance in nearly 50% of highly active patients 6 months after ACLR. Squat and plyometric jumps involving the injured leg pose the greatest challenge to subjects, suggesting that insufficiently recovered knee extensor strength, also evidenced by isokinetic tests, represents a key functional deficit that requires particular attention. Isokinetic strength tests further showed that knee extensor strength deficits were more pronounced when reconstruction was performed with QT autografts, whereas knee flexor strength was more strongly affected when SGT grafts were used. Moreover, BIA test results were significantly affected by age and sex, with elderly men showing a slower recovery. Future research should aim to elucidate the pathophysiological reasons underlying the persistent weakness of the knee extensor muscles, with the aim to develop more effective training interventions.

## Figures and Tables

**Table 1:** Sample characteristics.

Sample Size	227
Age (yrs)	23.8 ± 8.4
Males:Females (%)	61.6:38.4
QT:SGT (%)	61.2:38.8
Primary reconstruction: Revision: Contralateral ACL surgery (%)	80.6:10.6:8.8
Simple ACL injury: Complex ACL injury (%)	48.5:51.5

ACL: Anterior cruciate ligament; QT: Quadriceps tendon graft; SGT: Semitendinosus/gracilis tendon graft. Complex injuries are characterized by the involvement of meniscal tears or chondral lesions.

**Table 2:** Patient-reported outcome measures.

	VAS		Lysholm		Tegner	
	Baseline	Δ 0–6 mo.	Baseline	Δ 0–6 mo.	Baseline	Δ 0–6 mo.
Total (*n* = 227)						
(*n* = 227)	0.86 ± 1.60	+0.21 ± 1.86	95.21 ± 12.23	−5.96 ± 13.60	7.38 ± 1.62	−0.37 ± 1.54
Graft (SGT: *n* = 88, QT: *n* = 139)						
SGT	0.89 ± 1.88	+0.18 ± 2.07	95.34 ± 14.60	−6.49 ± 15.03	7.17 ± 1.72	−0.32 ± 1.56
QT	0.85 ± 1.39	+0.23 ± 1.73	95.12 ± 10.53	−5.63 ± 12.69	7.52 ± 1.54	−0.39 ± 1.53
Age group (10–19 yrs: *n* = 88, 20–29 yrs: *n* = 105, 30–50 yrs: *n* = 34)						
10–19 yrs	0.90 ± 1.78	+0.03 ± 1.77	93.78 ± 16.42	−2.66 ± 14.70 *	7.78 ± 1.47	−0.17 ± 1.16
20–29 yrs	0.79 ± 1.49	+0.39 ± 1.75	96.32 ± 8.30	−7.51 ± 11.75	7.53 ± 1.39	−0.55 ± 1.63
30–50 yrs	1.00 ± 1.46	+0.15 ± 2.40	95.41 ± 9.53	−9.59 ± 14.84	5.92 ± 1.83 *	−0.30 ± 2.02
Sex (Men: *n* = 144, Women: *n* = 83)						
Men	0.94 ± 1.63	+0.17 ± 1.97	94.76 ± 13.75	−5.73 ± 15.54	7.52 ± 1.63	−0.43 ± 1.71
Women	0.72 ± 1.53	+0.29 ± 1.67	95.99 ± 9.07	−6.34 ± 9.64	7.15 ± 1.57	−0.26 ± 1.20

Visual analog scale pain (VAS), Lysholm, and Tegner scores (as measured before injury (baseline)) and changes until the 6-months follow-up (Δ 0–6 mo.). Values highlighted with * are significantly different from the other respective subgroups (*p* < 0.05).

**Table 3:** Classifications of Back in Action test results.

	Very Poor (%)	Poor (%)	Norm (%)	Good (%)	Very Good (%)
2-leg stability	2 (0.8)	8 (3.3)	71 (29.0)	**42 (17.1)**	122 (49.8)
1-leg stability H	6 (2.4)	9 (3.7)	90 (36.7)	**64 (26.1)**	76 (31.0)
1-leg stability I	7 (2.9)	11 (4.5)	91 (37.1)	**50 (20.4)**	86 (35.1)
2-leg jump height	20 (8.2)	15 (6.1)	71 (29.0)	**27 (11.0)**	112 (45.7)
2 jump power	2 (0.8)	6 (2.4)	94 (38.4)	**37 (15.1)**	106 (43.3)
1-leg jump height H	19 (7.8)	9 (3.7)	91 (37.1)	**24 (9.8)**	102 (41.6)
1-leg jump power H	5 (2.0)	4 (1.6)	44 (18.0)	47 (19.2)	**145 (59.2)**
1-leg jump height I	38 (15.5)	22 (9.0)	**79 (32.2)**	28 (11.4)	78 (31.8)
1-leg jump power I	6 (2.4)	6 (2.4)	65 (26.5)	37 (15.1)	**131 (53.5)**
Plyometric RSI	23 (9.4)	31 (12.7)	**154 (62.9)**	21 (8.6)	16 (6.5)
Speedy healthy	19 (7.8)	16 (6.5)	83 (33.9)	**53 (21.6)**	74 (30.2)
Speedy injured	19 (7.8)	16 (6.5)	**89 (36.3)**	59 (24.1)	62 (25.3)
Quick feet	22 (9.0)	5 (2.0)	75 (30.6)	**45 (18.4)**	98 (40.0)

Bold figures reflect the median test results, cells highlighted in gray show the respective interquartile ranges. In single-leg tests, H and I denominate the healthy and injured leg, respectively. RSI is the reactive strength index.
